# Genetic variant in fat mass and obesity-associated gene associated with type 2 diabetes risk in Han Chinese

**DOI:** 10.1186/1471-2156-14-86

**Published:** 2013-09-22

**Authors:** Yun Qian, Sijun Liu, Feng Lu, Huizhang Li, Meihua Dong, Yudi Lin, Jiangbo Du, Yuan Lin, Jianhang Gong, Guangfu Jin, Juncheng Dai, Zhibin Hu, Hongbing Shen

**Affiliations:** 1 Department of Chronic Non-communicable Disease Control, Wuxi Center for Disease Control and Prevention, Wuxi, China; 2 Department of Epidemiology and Biostatistics, MOE Key Laboratory of Modern Toxicology, School of Public Health, Nanjing Medical University, Nanjing, China; 3 Department of Chronic Non-communicable Disease Control, Zhejiang Provincial Center for Disease Control and Prevention, Hangzhou, China; 4 Department of Cancer Prevention and Treatment, Zhejiang Cancer Hospital, Hangzhou, China; 5 State Key Laboratory of Reproductive Medicine, Nanjing Medical University, Nanjing, China

**Keywords:** Fat mass and obesity-associated Gene, Genome-wide association study, Genetic susceptibility, Single nucleotide polymorphism, Type 2 diabetes

## Abstract

**Background:**

Genome-wide association study (GWAS) has identified that rs8050136 *C/A* polymorphism in fat mass and obesity-associated gene (*FTO*) was associated with the risk of type 2 diabetes (T2D) in Europeans. But this association was abolished after adjustment for body mass index (BMI), suggesting that the effect of rs8050136 on T2D risk might be mediated by BMI in Europeans. However, the findings in subsequent studies were inconsistent among Asian populations. To determine whether rs8050136 polymorphism in *FTO* is independently associated with the risk of T2D in Chinese, we conducted a case–control study with 2,925 T2D patients and 3,281 controls in Han Chinese.

**Results:**

Logistic regression revealed that the *A* allele of rs8050136 was significantly associated with an increased risk of T2D, independent of BMI (odds ratio (OR) = 1.17, 95% confidence interval (95% CI) = 1.03-1.32, p = 0.016). Meta-analysis containing 10 reported studies and our data with a total of 15,819 cases and 18,314 controls further confirmed the association between rs8050136 polymorphism and T2D risk in East Asians (OR = 1.13, 95% CI = 1.07-1.19).

**Conclusions:**

Our findings indicate that the genetic variant in *FTO* may contribute to T2D risk in Han Chinese and rs8050136 polymorphism may be a genetic marker for T2D susceptibility.

## Background

Type 2 diabetes (T2D) is a major global health problem with an increasing prevalence over the world [1]. More than 60% of the world’s diabetes patients will be Asians in 2025 [2]. In China, it has become an epidemic concern due to the western lifestyle [3]. Although the etiology of T2D has not been well elucidated, genetic and environmental factors are considered to be involved in its development [4].

In 2007, genome-wide association studies (GWAS) firstly reported that single nucleotide polymorphism (SNP) rs8050136 in fat mass and obesity-associated gene (*FTO*) was associated with the risk of T2D in European populations [5,6]. However, this association was not significant after adjustment for body mass index (BMI). Another GWAS identified SNP rs9939609 in *FTO* as strongly associated with both BMI and obesity in Europeans [7]. Based on the public HapMap SNP database, rs8050136 and rs9939609 were in the perfect linkage disequilibrium (LD) (r^2^ = 1). Therefore, it is suggested that the effect of rs8050136 or rs9939609 on T2D risk may be mediated through a primary effect on obesity in European populations.

Following the initial reports in populations of European descent, Horikoshi et al. found that rs8050136 was also associated with T2D risk in a Japanese population, however, which was independent of BMI [8]. In contrast, Ng et al. did not find a significant association in Hongkong and Korean populations (OR = 1.09, 95% CI = 0.97-1.23) [9]. Among Chinese populations, the association between rs8050136 and T2D risk failed to be replicated in Hu’s study [10]; however, Han et al. detected a positive association in another Chinese Han population [11]. To determine whether rs8050136 polymorphism in *FTO* is independently associated with the risk of T2D, we conducted a case–control study with 2,925 T2D cases and 3,281 controls in Han Chinese. In addition, we also undertook a systematic meta-analysis including 15,819 cases and 18,314 controls to assess the association between this *FTO* polymorphism and T2D risk in East Asians.

## Results

The distributions of demographic and selected clinical characteristics of 2,925 T2D cases and 3,281 controls were summarized in (Additional file 1: Table S1). No significant differences were observed in the distributions of sex, smoking and drinking status. Type 2 diabetes cases were older than controls and had significantly higher levels of BMI, fasting blood glucose (FBG), triglyceride (TG), total cholesterol (TC), blood pressure and significantly lower level of high-density lipoprotein cholesterol (HDL-C). The genotype distribution of rs8050136 in *FTO* and its association with T2D risk between the T2D cases and controls were shown in Table 1. The observed genotype frequencies for rs8050136 were in Hardy–Weinberg equilibrium (HWE) in the controls (p = 0.941). Significant differences of genotype distribution between the T2D cases and controls were observed for rs8050136 in the dominant model (crude OR = 1.30, 95% CI = 1.15-1.46, p = 3.0×10^-5^) and the additive model (crude OR = 1.27, 95% CI = 1.13-1.41, p = 3.8×10^-5^). With the adjustment for age, sex and BMI in the logistic regression model, rs8050136 *CA/AA* genotypes were associated with a significantly increased risk of T2D (OR = 1.19, 95% CI = 1.04-1.37, p = 0.011), as compared with *CC* genotype. Similar association was also observed in the additive model (adjusted OR = 1.17, 95% CI = 1.03-1.32, p = 0.016).

**Table 1:** The genotype distribution of rs8050136 and the association with type 2 diabetes risk in Han Chinese

**Genotype**	**Cases N (%)**	**Controls N (%)**	**OR (95% CI)**	**p-valu*e***	**OR (95% CI)**	**p-valu*e***
*CC*	2205 (76.1)	2625 (80.5)	1.00		**1.00**	
*CA*	652 (22.5)	602 (18.5)	1.29(1.14–1.46)	6.3×10^-5^	**1.20(1.04–1.38)**	**0.011**
*AA*	41 (1.4)	35 (1.1)	1.39(0.89–2.20)	0.152	**1.11(0.66–1.87)**	**0.687**
*CA*/*AA*	693 (23.9)	637 (19.5)	1.30(1.15–1.46)	3.0×10^-5^	**1.19(1.04–1.37)**	**0.011**
Additive			1.27(1.13–1.41)	3.8×10^-5^	**1.17(1.03–1.32)**	**0.016**

Genotype was successfully detected in 2,898 T2D cases and 3,262 controls. Odds ratios (95% confidence intervals) and p-values were calculated in the logistic regression model. Results of adjustment for age, sex and body mass index are in bold.

We then conducted the stratified analyses by age, sex and BMI. As shown in Table 2, the associations between rs8050136 polymorphism and T2D risk were also evident in the groups of elderly subjects (age > 56 years: OR = 1.24, 95% CI = 1.05-1.46, p = 0.010), female subjects (OR = 1.20, 95% CI = 1.02-1.40, p = 0.025) and those with normal weight (BMI < 24 kg/m^2^: OR = 1.25, 95% CI = 1.07-1.46, p = 0.005). No heterogeneity was observed among these subgroups.

**Table 2:** The stratified analyses on the association between rs8050136 and the risk of type 2 diabetes

**Variables**	**Cases**	**Controls**	**OR (95% CI)**	**p-valu*e***	**p-valu*e***
Age					
≤56	863/246/15	1165/260/17	1.07(0.88–1.30)	0.484	**0.258**
>56	1341/406/26	1460/342/18	1.24(1.05–1.46)	0.010	
Sex					
females	1382/400/28	1641/372/23	1.20(1.02–1.40)	0.025	**0.557**
males	823/252/13	984/230/12	1.11(0.90–1.36)	0.350	
BMI					
normal	849/255/10	2107/471/28	1.25(1.07–1.46)	0.005	**0.663**
overweight	945/255/18	438/111/5	1.10(0.88–1.39)	0.391	
obesity	394/137/12	74/20/2	1.19(0.75–1.89)	0.454	

Listed are the counts of *CC/CA/AA* genotypes in T2D cases and controls. Odds ratios (95% confidence intervals) and p-values were calculated in the logistic regression model with adjustment for age, sex and body mass index where appropriate. P-value in bold is for the heterogeneity test based on chi-squared based Q test.

**Figure 1:**
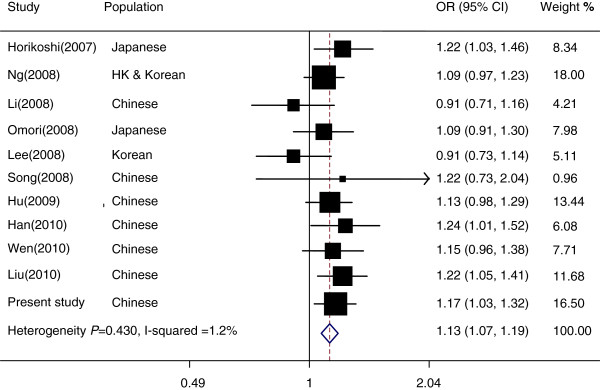
**Meta-analysis of the association between rs8050136 polymorphism and the risk of T2D in East Asians.** To estimate the pooled effect size of SNP rs8050136 on T2D risk in East Asians, a meta-analysis including 10 published studies on the association between rs8050136 polymorphism and T2D risk in East Asians and our present study was conducted. There was no heterogeneity of ORs across studies (p = 0.430 and I^2^=1.2%). The pooled OR for SNP rs8050136 and the risk of T2D was significant (pooled OR = 1.13, 95% CI = 1.07-1.19) in East Asians.

**Figure 2:**
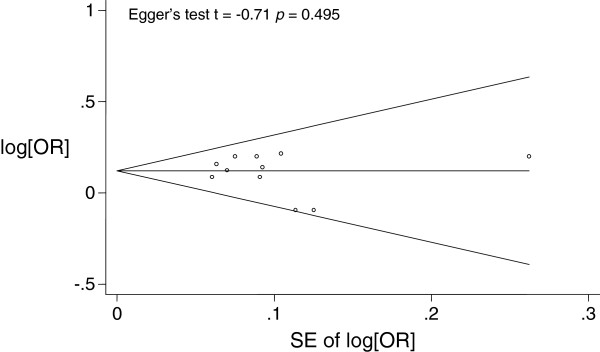
**Funnel plot analysis to detect the publication bias in East Asians.** The funnel plot was symmetrical and the result from Egger’s test was non-significant (t = −0.71, p = 0.495).

A total of 10 published studies on the association between rs8050136 polymorphism and T2D risk in East Asian populations were summarized in (Additional file 2: Table S2) [8-17]. These 10 studies, together with our present study, were included in the meta-analysis. Overall meta-analysis results revealed that rs8050136 *A* allele was associated with an increased T2D risk in East Asians (OR = 1.13, 95% CI = 1.07-1.19, Figure 1). Further meta-analysis also confirmed the association with T2D risk in Chinese Hans (OR = 1.16, 95% CI = 1.08-1.23) (Additional file 3: Figure S1). No obvious heterogeneity was observed between the selected studies (heterogeneity p = 0.430 and I^2^ = 1.2% for East Asians; heterogeneity p = 0.569 and I^2^ = 0.0% for Chinese Hans). Funnel plot and Egger’s test were used to address the potential publication bias and no significant publication bias was found (t = −0.71, p = 0.495 for East Asians, Figure 2; t = −0.53, p = 0.617 for Chinese Hans, Additional file 4: Figure S2).

## Discussion

In this study, we investigated the association of rs8050136 polymorphism in *FTO* with T2D risk in Han Chinese. We found that rs8050136 *A* allele was significantly associated with an increased T2D risk in Han Chinese, which was further supported by a meta-analysis containing 10 reported studies in East Asians and our present study.

Although a number of studies have investigated the association between *FTO* rs8050136 polymorphism and T2D risk in Asian populations, the results were inconsistent. Horikoshi et al. reported a significant association (OR = 1.22, 95% CI = 1.03-1.46, p = 0.025) between rs8050136 and T2D risk in 864 cases and 864 controls in Japanese [8], which was supported by a larger study comprising 1,912 cases and 2,041 controls in a Han Chinese population (OR = 1.22, 95% CI= 1.05-1.41, p = 0.008) [14]. However, Omori et al. were not able to replicate the association (OR = 1.09, 95% CI = 0.91-1.30, p = 0.350) in a case–control study with 1,630 T2D cases and 1,064 controls in Japanese [12]. In the present study, we observed the significant association between rs8050136 and T2D risk (OR = 1.17, 95% CI = 1.03-1.32, p = 0.016) after adjustment for age, sex and BMI. The discrepancy of the association of rs8050136 with T2D risk in East Asians may be due to the relatively low frequency (minor allele frequencies (MAFs) = 0.12 and 0.17 for Chinese and Japanese populations) leading to a low statistical power, as compared with the populations of European descent (MAF = 0.45). Therefore, the meta-analysis conducted in this study may represent a powerful approach to clarify the relationship between rs8050136 and T2D risk by pooling 15,819 cases and 18,314 controls and confirm this association in East Asians.

Our study showed the *A* allele of rs8050136 was significantly associated with an increased risk of T2D, independent of BMI. A recent meta-analysis including 96,551 East and South Asians reported that the minor allele for the rs9939609 *FTO* SNP (perfect LD with rs8050136) significantly increased the risk of both obesity and T2D, and the association between the *FTO* locus and risk of T2D remained significant after adjustment for BMI [18]. Interestingly, a recent meta-analysis of 41,504 Scandinavians revealed that the significant association for rs9939609 with the risk of T2D also remained after correction for BMI [19]. These results were not consistent with those of the earlier study that showed the association of rs8050136 with T2D risk was mediated through BMI among Europeans [6].

The SNP rs8050136 is mapped to the intron 1 of *FTO* gene on chromosome 16. The exact function of the gene product of *FTO* is not yet understood and how the polymorphism affects the risk of T2D is unclear. Based on sequence homology, *FTO* gene is predicted to encode a 2-oxoglutarate-dependent demethylase enzyme, which affects nucleic acid demethylation [20]. This gene may be critical in epigenetic regulation of the progress of T2D. Recently, Bravard et al. found a significant increase of *FTO* mRNA and protein in skeletal muscle of T2D patients, suggesting that *FTO* may involve in T2D pathogenesis through oxidative metabolism, lipogenesis and oxidative stress in muscle defects [21].

The limitation of this case–control study was that most of the T2D cases were diagnosed before the surveys and therefore, their BMI may not be a perfect measure of adiposity, particularly in lean individuals and in East Asians who usually have more abdominal fat and less muscle mass than Caucasians. Although we adjusted BMI for adiposity in detecting the association of *FTO* polymorphism with T2D risk, we cannot rule out the possibility of residual confounding of obesity, because the alternative measures (such as DEXA fat mass) of adiposity were not available in this study. More alternative measures of adiposity need to be considered in future studies.

## Conclusions

In summary, we evaluated the association of *FTO* rs8050136 polymorphism with the risk of T2D in a large case–control study and 2 meta-analyses and confirmed that the *A* allele was significantly associated with an increased risk of T2D in East Asians. Further studies are warranted to clarify the potentially biological mechanisms of *FTO* rs8050136 polymorphism in T2D pathogenesis.

## Methods

### Study subjects

All subjects were genetically unrelated ethnic Han Chinese and the study population was previously described [22,23]. In brief, 2,925 T2D cases and 3,281 controls were recruited from 2 community-based chronic non-communicable disease surveys in Jiangsu province, from 2004 to 2008. Subjects were considered to have T2D if they had a history of T2D or if their fasting glucose was 7.0 mmol/l or higher. Those without history of diabetes, hypertension, coronary heart disease, stroke, cancer and with fasting glucose < 5.6 mmol/l were selected as controls from the same population and frequency-matched to the cases by age, sex and residential area. All participants were interviewed by trained interviewers to obtain information on demographic data, smoking, alcohol drinking, family history, physical activity and disease history using standard questionnaires. At the same time, anthropometrical measurements were performed. A 5 ml blood sample was collected from each subject after fasting for more than 8 hours. FBG and lipid levels, including TC, TG, and HDL-C were detected in standard methods. According to the criteria proposed by the Working Group on Obesity in China, we defined overweight as 24 kg/m^2^ ≤ BMI < 28 kg/m^2^, and obesity as BMI ≥ 28 kg/m^2^[24]. The distributions of demographic and clinical characteristics of the T2D cases and controls were summarized in (Additional file 1: Table S1). This study was approved by the institutional review board of Nanjing Medical University. Written informed consent was obtained from each participant.

### Genotyping assay

Genomic DNA was isolated from leukocyte pellets of venous blood by proteinase K digestion, followed by phenol-chloroform extraction. TaqMan OpenArray Genotyping System (Life Technologies, Carlsbad, CA, USA) and the iPLEX Sequenom MassARRAY platform (Sequenom, Inc., San Diego, CA, USA) were used to perform genotyping. Genotype was successfully detected in 2,898 cases and 3,262 controls, with the call rate being 99.2%. Genotyping was performed by technicians without knowing the statuses of T2D cases or controls. Two blank controls in each plate were used for quality control. 96 randomly selected duplicated samples were examined on two platforms and the concordant rate was 100%.

### Meta-analysis

We searched all case–control studies on the association between *FTO* gene rs8050136 polymorphism and T2D risk in East Asian populations. Eligible studies, published in English, up to August, 2012, were identified by using the search terms “*FTO* and polymorphism and type 2 diabetes” in PubMed. Studies that were not conducted in East Asians were excluded in manual. ORs and 95% CIs were calculated in additive model after adjustment in logistic regression. Cochran’s χ^2^-based Q-statistic test was performed to assess the heterogeneity among the included studies. The combined OR was calculated using the Mantel–Haenszel method in fixed-effect model with 95% CI in Woolf’s method as there was no significant heterogeneity. Publication bias was evaluated with Egger’s *t* test.

### Statistical analyses

The differences in demographic variables, selected variables, and frequencies of the genotypes between cases and controls were evaluated by using the Student’s *t*-test for continuous variables and the χ^2^ - test for categorical variables. The Hardy-Weinberg equilibrium was tested by a goodness-of-fit χ^2^ - test in controls. The association between SNP and T2D risk was estimated by computing the ORs and 95% CIs using unconditional logistic regression analysis after adjustment for age, sex and BMI. Heterogeneity test for stratified analyses of the selected variables was conducted with the χ^2^-based Q test. Multivariate linear regression analyses were performed to test the correlation between the genotypes and quantitative BMI trait after adjustment for age and sex. The statistical power was calculated by PS (power and sample size calculation) 3.0.2 software. All statistical analyses were performed by using STATA version 11.0 (StataCorp LP, College Station, TX, USA).

## Abbreviations

GWAS: Genome-wide association study; FTO: Fat mass and obesity-associated gene; T2D: Type 2 diabetes; BMI: Body mass index; OR: Odds ratio; 95% CI: 95% confidence interval; SNP: Single nucleotide polymorphism; LD: Linkage disequilibrium; FBG: Fasting blood glucose; TC: Total cholesterol; TG: Triglyceride; HDL-C: High-density lipoprotein-cholesterol; HWE: Hardy–weinberg equilibrium; MAF: Minor allele frequency.

## Competing interests

The authors declare that they have no competing interests.

## Authors’ contributions

YQ carried out the molecular genetic studies, participated in the genotype detection and statistical analysis, drafted the manuscript and is the corresponding author. SL and FL performed the experiments and analyzed the data. HL participated in the genotype detection and the statistical analysis. MD and YL participated in the field investigation. JD, JG and YL participated in the genotype detection. GJ and JD participated in the design and the data analysis. ZH involved in the design and coordination. HS conceived the study, edited the manuscript, and is the corresponding author. All authors read and approved the final manuscript.

## Supplementary Material

Additional file 1: Table S1 General characteristics of type 2 diabetes cases and controls. This table shows the characteristics of the study population. No significant differences were observed in the distributions of sex, smoking and drinking status. Type 2 diabetes cases were older than controls and had significantly higher levels of body mass index, fasting blood glucose, triglyceride, total cholesterol, blood pressure and significantly lower level of high-density lipoprotein cholesterol. Click here for file

Additional file 2: Table S2 Published studies on the association between rs8050136 polymorphism and the risk of type 2 diabetes in East Asians. The table displays minor allele frequencies (MAFs), sample sizes, adjusted odds ratios (ORs) and 95% confidence intervals (CIs), p-values, populations, journals, authors and published year of the 10 published studies among East Asian populations. They were included in the meta-analyses together with the present study. Click here for file

Additional file 3: Figure S1 Meta-analysis of the association of rs8050136 polymorphism with the risk of type 2 diabetes in Han Chinese. The figure shows the results of the meta-analysis covering 6 reported studies in Chinese Han populations and our present study. Click here for file

Additional file 4: Figure S2 Funnel plot analysis to detect the publication bias in Chinese Han populations. The figure shows the results of the funnel plot analysis to address the potential publication bias about the 7 studies conducted among Chinese Han populations. Click here for file
