# Supplementary material for: Genetic variant in fat mass and obesity-associated gene associated with type 2 diabetes risk in Han Chinese
Source: BMC Genet. 2013 Sep 22;14:86. doi: 10.1186/1471-2156-14-86 (PMC3848839; doi:10.1186/1471-2156-14-86)
Supplement: Additional file 1: Table S1 — General characteristics of type 2 diabetes cases and controls. This table shows the characteristics of the study population. No significant differences were observed in the distributions of sex, smoking and drinking status. Type 2 diabetes cases were older than controls and had significantly higher levels of body mass index, fasting blood glucose, triglyceride, total cholesterol, blood pressure and significantly lower level of high-density lipoprotein cholesterol. [file 1471-2156-14-86-S1.doc]

**Table S1 General characteristics of type 2 diabetes cases and controls.**

| Variables ^a^ | Cases  N=2925 (%) | Controls N=3281 (%) |
| --- | --- | --- |
| Age | 58.21±10.11 | 56.57±9.88 |
| ≤56 | 1138((38.9) | 1451(44.2) |
| ＞56 | 1786(61.1) | 1830(55.8) |
| Sex |  |  |
| Females | 1827(62.5) | 2047(62.4) |
| Males | 1098(37.5) | 1234(37.6) |
| Smoking status |  |  |
| No | 2185(75.3) | 2431(74.6) |
| Yes | 717(24.7) | 827(25.4) |
| Drinking status |  |  |
| No | 2345(81.5) | 2619(80.5) |
| Yes | 534(18.5) | 635(19.5) |
| Body mass index (kg/m^2^) | 25.05±3.50 | 22.12±2.63 |
| ＜24 | 1124(38.7) | 2620(80.0) |
| 24-28 | 1232(42.5) | 559(17.1) |
| ≥28 | 546(18.8) | 96(2.9) |
| Fasting blood glucose (mmol/l) | 9.03±3.52 | 4.52±0.53 |
| Triglyceride (mmol/l) | 2.57±2.47 | 1.09±0.45 |
| Total cholesterol (mmol/l) | 4.98±1.44 | 4.39±0.80 |
| High-density lipoprotein cholesterol (mmol/l) | 1.46±0.48 | 1.62±0.38 |
| Systolic blood pressure (mmHg) | 135.47±20.99 | 117.68±14.17 |
| Diastolic blood pressure (mmHg) | 80.36±10.87 | 74.14±9.24 |

Data are showed as means ± SDs or numbers (percentages).

^a^ There were missing data for age (1 subject), smoking (46 subjects), drinking (73 subjects), body mass index (29 subjects), fasting blood glucose (24 subjects), triglyceride (21 subjects), total cholesterol (21 subjects), high-density lipoprotein cholesterol (21 subjects), systolic blood pressure (7 subjects) and diastolic blood pressure (8 subjects).
