# Supplementary material for: Genetic variant in fat mass and obesity-associated gene associated with type 2 diabetes risk in Han Chinese
Source: BMC Genet. 2013 Sep 22;14:86. doi: 10.1186/1471-2156-14-86 (PMC3848839; doi:10.1186/1471-2156-14-86)
Supplement: Additional file 2: Table S2 — Published studies on the association between rs8050136 polymorphism and the risk of type 2 diabetes in East Asians. The table displays minor allele frequencies (MAFs), sample sizes, adjusted odds ratios (ORs) and 95% confidence intervals (CIs), p-values, populations, journals, authors and published year of the 10 published studies among East Asian populations. They were included in the meta-analyses together with the present study. [file 1471-2156-14-86-S2.doc]

**Table S2 Published studies on the association between rs8050136 polymorphism and the risk of type 2 diabetes in East Asians.**

| No. | Author | Journal | Year | Sample size (case/control) | Population | MAF | OR(95%CI)_add_ | *P* | Adjusted |
| --- | --- | --- | --- | --- | --- | --- | --- | --- | --- |
| 1 | Horikoshi [8] | Diabetologia | 2007 | 864/864 | Japanese | 0.200 | 1.22(1.03-1.46) | 0.025 | age, sex, BMI |
| 2 | Ng [9] | Diabetes | 2008 | 3041/3687 | HK & Korean | 0.118 | 1.09(0.97-1.23) | 0.130 | age, sex, BMI |
| 3 | Li [13] | Diabetes | 2008 | 424/2786 | Chinese | - | 0.91(0.71-1.16) | 0.430 | age, sex, BMI |
| 4 | Omori [12] | Diabetes | 2008 | 1630/1064 | Japanese | 0.192 | 1.09(0.91-1.30) | 0.350 | age, sex, BMI |
| 5 | Lee [15] | J Hum Genet | 2008 | 908/502 | Korean | 0.140 | 0.89(0.70-1.14) | 0.347 | age, sex, BMI |
| 6 | Song [16] | Obesity | 2008 | 77/163 | Chinese | - | 1.22(0.73-2.04) | - | BMI & others |
| 7 | Hu [10] | PLoS One | 2009 | 1849/1785 | Chinese | 0.118 | 1.13(0.98-1.29) | 0.146 | age, sex, BMI |
| 8 | Han [11] | BMC Med Genet | 2010 | 1024/1005 | Chinese | 0.110 | 1.24(1.01-1.52) | 0.041 | age, sex, BMI |
| 9 | Wen [17] | PLoS One | 2010 | 1165/1136 | Chinese | 0.119 | 1.15(0.96-1.38) | 0.140 | age, sex, BMI |
| 10 | Liu [14] | Obesity | 2010 | 1912/2041 | Chinese | 0.117 | 1.22(1.05-1.41) | 0.008 | age, sex, BMI |
| 11 | Present study |  |  | 2925/3281 | Chinese | 0.101 | 1.17(1.03-1.32) | 0.016 | age, sex, BMI |
