# Supplementary material for: Genetic variant in fat mass and obesity-associated gene associated with type 2 diabetes risk in Han Chinese
Source: BMC Genet. 2013 Sep 22;14:86. doi: 10.1186/1471-2156-14-86 (PMC3848839; doi:10.1186/1471-2156-14-86)
Supplement: Additional file 3: Figure S1 — Meta-analysis of the association of rs8050136 polymorphism with the risk of type 2 diabetes in Han Chinese. The figure shows the results of the meta-analysis covering 6 reported studies in Chinese Han populations and our present study. [file 1471-2156-14-86-S3.doc]

Heterogeneity p = 0.569,

I-squared = 0.0%

Liu (2010)

Wen (2010)

Hu (2009)

Song (2008)

Han (2010)

Present study

Study

Li (2008)

1.16 (1.08, 1.23)

1.22 (1.05, 1.41)

1.15 (0.96, 1.38)

1.22 (0.73, 2.04)

1.17 (1.03, 1.32)

1.24 (1.01, 1.52)

1.13 (0.98, 1.29)

OR (95% CI)

0.91 (0.71, 1.16)

100.00

19.28

12.73

1.59

27.23

10.03

22.19

Weight %

6.95

1

.49

1

2.04

**Figure S1. Meta-analysis of the association of rs8050136 polymorphism with the risk of type 2 diabetes in Han Chinese.**
