# Supplementary material for: Genetic variant in fat mass and obesity-associated gene associated with type 2 diabetes risk in Han Chinese
Source: BMC Genet. 2013 Sep 22;14:86. doi: 10.1186/1471-2156-14-86 (PMC3848839; doi:10.1186/1471-2156-14-86)
Supplement: Additional file 4: Figure S2 — Funnel plot analysis to detect the publication bias in Chinese Han populations. The figure shows the results of the funnel plot analysis to address the potential publication bias about the 7 studies conducted among Chinese Han populations. [file 1471-2156-14-86-S4.doc]

**Figure S2.** **Funnel plot analysis to detect the publication bias in Chinese Han populations.**

Egger’s test t=-0.53, *P*=0.617

logOR

SE of logOR

0

.1

.2

.3

-.5

0

.5

1
